# The Impact of COVID-19 on the Language Skills of Preschool Children: Data from a School Screening Project for Language Disorders in Greece

**DOI:** 10.3390/children12030376

**Published:** 2025-03-18

**Authors:** Eleni Kyvrakidou, Giannis Kyvrakidis, Anastasia S. Stefanaki, Asterios Asimenios, Athanasios Gazanis, Asterios Kampouras

**Affiliations:** 1 Special Treatment Center ‘Nous & Logos’, 554 38 Thessaloniki, Greece; nous.kai.logos@gmail.com (E.K.); john_kivra@hotmail.com (G.K.); asteriosasimenios@yahoo.com (A.A.); 2 Chair of Social Pediatrics, TUM School of Medicine, Technical University of Munich, 81377 Munich, Germany; stefanakianast@gmail.com; 3 Psychiatry Department, 424 General Military Hospital, 564 29 Thessaloniki, Greece; gazanis@hotmail.com; 4 4th Pediatric Department, Papageorgiou Hospital, Aristotle University of Thessaloniki, 541 24 Thessaloniki, Greece

**Keywords:** COVID-19, pandemic, quarantine, children, preschool, language development, language skills, screening for language disorders, words and pseudowords, means of expression

## Abstract

**Background/Objectives:** The COVID-19 pandemic has significantly affected children’s lives, particularly preschool-aged children who undergo rapid biological and psychosocial development. This study aimed to investigate the effects of the COVID-19 pandemic on the language skills of preschool children in Greece. **Methods:** To that end, a widely used screening tool was applied in a screening project involving 213 preschoolers. Language skills were assessed in three groups of children aged 2–4 years old before, during and after the pandemic. **Results:** A significant increase in the number of children with atypical language skills profile was identified in relation to the preschoolers after the pandemic versus those before or during the pandemic period. A higher prevalence of atypical profiles was observed in girls than in boys. Interestingly, an increase in the number of successfully produced or repeated words and pseudowords, along with enhanced expressive abilities, was observed during the pandemic compared to the periods before and after. **Conclusions:**Our findings suggest that post-pandemic preschool children exhibit higher rates of atypical language skill profiles compared to those assessed before and during the pan-demic. Given the importance of language development as a critical aspect of children’s overall personality and well-being, further research is needed to explore the impact of specific pandemic-related factors on language competency. These factors include mask-wearing, increased screen time, reduced social interaction and exposure to language-rich environments, as well as impaired mental health and parental distress. Additionally, personalized interventions should be developed to support healthier developmental outcomes.

## 1. Introduction

The potential impact of the COVID-19 pandemic and the social distancing measures on the social and psycho-emotional development of children, particularly those of preschool age, continues to attract significant research interest worldwide [1]. Notably, longitudinal research on early adolescents has shown that the pandemic and social distancing measures had limited long-term effects on their mental health and development, which may be due to their greater neurocognitive resilience [2,3]. Conversely, the preschool age is a critical period of neurodevelopment characterized by extreme sensitivity to linguistic input, environmental variations and a near-total dependence on parents [4].

During the COVID-19 pandemic, social isolation, school closures, disruption of daily routines (such as sleep and physical activities), increase in passive screen time and the parents’ distress due to the broader effects of the lockdowns unsurprisingly appear to have affected young children’s emotions and behavior [4,5,6,7,8,9]. In terms of cognitive and learning skills, the responsibility for fostering improvement during this sensitive phase of personal growth and societal instability fell largely on the children themselves and their families; “low-achievers” or those with less-educated parents appeared to have had visibly poorer outcomes [10,11,12]. It is estimated that the stringent lockdown measures led to a reduction in educational attainment equivalent to between 0.3 and 1.1 years of schooling [5]. Apparently, the children most affected by the lockdowns in regards to their pro-social and emotional functions were those with special needs or requiring therapies such as speech, cognitive/behavioral, or psychoeducational therapy, like those with neurodevelopment disorders [8,13,14]. This could be attributed to both the nature of their conditions, which often involve difficulty with routine changes, and the disruption of their therapeutic plans [8,12,13,15]. Notably, emotional and behavioral issues were found to co-exist with language weaknesses [12].

However, even children with typical neurodevelopment may have faced delays or changes in language acquisition due to reduced social interaction and altered learning environments [16]. Against this background, this study focuses on preschool-aged children in Greece and investigates children with both typical and atypical language skills, providing a comprehensive view of how the COVID-19 pandemic and the imposed restrictive measures may have influenced children’s linguistic skills.

Our study aimed to address the following research questions:

First, whether rates of atypical language skills differed before, during and after the pandemic restrictions. Based on previous research, we hypothesized that the prevalence of atypical language skills would be higher in children assessed during or after the pandemic compared to those assessed before [16,17,18,19,20].

Second, we explored potential gender-related differences in the impact of the pandemic on children’s language skills. Prior research suggests that girls usually outperform boys in early language development, and, therefore, we anticipated that the language skills of the enrolled girls would be affected less severely than those of the enrolled boys by the pandemic [21]. By addressing these questions, our study seeks to contribute to the understanding of how pandemic-related restrictive measures affected preschool children’s language skills and, consequently, their overall linguistic development.

## 2. Materials and Methods

### 2.1. Study Sample and Ethics

This retrospective cohort study encompassed a total sample of 213 preschool-aged children, comprising 104 boys and 109 girls, with a mean age of 52 months (age range from 35 to 77 months). Most of the test responses were collected after the COVID-19 pandemic (47.4%), while the rest were collected before (29.1%) and during (23.4%) it. The sample was drawn from both urban and rural areas of Western and Central Macedonia, Greece, as part of a language disorder screening program conducted in schools by two speech and language therapists. The testing took place in preschool units during regular lesson hours, with the children’s legal caregivers present in a non-intrusive manner. The therapists independently observed and documented the children’s responses to the structured questionnaire, which will be detailed below. No video recordings were made during the assessment.

Prior to their enrollment, all children provided verbal consent after receiving an explanation of the purpose and procedure, tailored to their age and level of comprehension. The team conducting this study, consisting of therapists and other relevant professionals, remained vigilant throughout the process, carefully monitoring any signs of emotional distress or a desire to discontinue the examination, particularly among younger children. If distress was observed, children and their caregivers had the right to withdraw at any time. Written informed consent was obtained from their legal caregivers. This study was conducted in accordance with the Declaration of Helsinki [22].

### 2.2. Language Skills Assessment Process

For the assessment of the language skills of the participants, the specialists utilized the “AνOμιΛο4” test, a Greek culturally adapted and validated version of the French ERTL4 test. The ERTL4 test stands for “Épreuve de Repérage des Troubles du Langage—4 ans” (Screening Test for Language Disorders—4 years). It is a language assessment designed for early detection of speech and language issues in young children. The test was independently administered in a standardized manner by two qualified speech and language therapists of the Special Treatment Center “Nous & Logos” (E.K. and G.K.) across the three-time points (before, during and after the pandemic). Both therapists received a priori specific training on the ERTL4 protocol to ensure consistency and reliability across all assessments, and adhered strictly to the established guidelines for the ERTL4 test irrespective of the time period. To optimize standardization, regular inter-rater variability checks were performed to confirm that the administration and scoring remained consistent throughout this study. Any inter-rater disagreement was resolved via consensus.

The “AνOμιΛο4” consists of questions focusing on testing children’s speech, language and voice mainly at the age of 3.6–4.9 years old. During the test (Supplementary Table S3), children were asked to repeat a series of words, pseudowords, phrases and sentences of increasing complexity with or without the complementary use of visual material (i.e., images used to help children produce the assessed words, pseudowords and sentences). Thereby, their phonological processing, working memory and overall language abilities were evaluated. The test results categorize the children into three language skills profile groups: 1st: Typical speech and language; 2nd: Requiring follow-up; and 3rd: Potential speech delays or disorders [23,24,25]. Based on the analysis of the test results, our study sample was categorized according to these three children’s profiles, with only the first profile defined as typical and both the second and third profiles classified as atypical [23].

Another classification of the sample was based on the timing of the evaluation concerning the enforcement of pandemic measures: before (2014–2019), during (2019–2020) and after (2020–2023) the COVID-19 pandemic. Different cohorts of children were assessed at each time point (before COVID-19 *n* = 62; during COVID-19 *n* = 50; and after COVID-19 *n* = 101).

As a secondary analysis of this study, the specialists measured the number of “words” and “pseudowords” produced by the participants as well as their “expression”. The number of words assessed includes age-related phonological combinations which are easily produced by children with typical language skills (e.g., banana, key, glasses, vase, home, snow and closet). The children were later asked to repeat a number of pseudowords based on a predefined list of phonetically plausible but non-existent words (e.g., Indian names including hofa, vlegkosa, siggi, vgoni, plouma, gousoma and tokepia) which were integrated into tasks designed to elicit spontaneous verbal output. The total count of pseudowords was determined based on transcription analysis by trained evaluators. Pseudowords serve as indicators of typical language skills, particularly in the areas of phonological processing, vocabulary acquisition and working memory.

Children’s expression was quantified using a composite score that considered both lexical diversity (the number of unique words used) and syntactic complexity (the variety and structure of sentences). This score was derived using a standardized rating scale, where higher values indicated greater fluency, coherence and grammatical complexity. Participants who articulated more than 8 “Words and Pseudowords”, with an “Expression” score exceeding 5 (as determined by the rating scale), were classified as children with typical language skills; those below this threshold were categorized as having atypical language skills [26,27].

### 2.3. Statistical Analysis

The data were encoded with Microsoft Excel, which was also used to produce the charts of this research. Nominal variables are presented with relative and absolute frequencies, while scale variables are presented with mean values (Μ) and standard deviations (SDs). The distributions of the variables are presented with bar graphs and histograms. Comparisons of mean values between three or more groups were conducted using the parametric one-way ANOVA test and with an additional post hoc analysis using the Bonferroni criterion. Furthermore, the chi-square test of independence was applied to investigate associations between nominal variables. The significance level for all tests was set to a = 0.05. All analyses were conducted through the Statistical Package for the Social Sciences software (SPSS v.25).

## 3. Results

**Figure 1:**
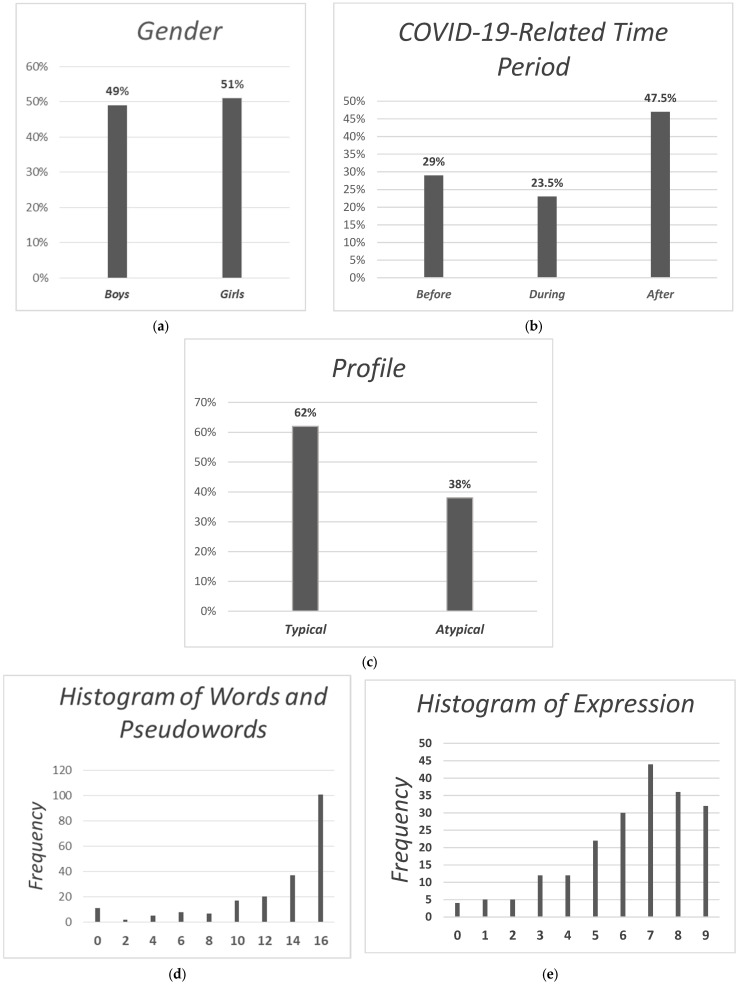
Descriptive statistics: (**a**) bar chart illustrating the distribution of the study sample by gender; (**b**) bar chart illustrating the distribution of the study sample by COVID-19 pandemic-related time periods; (**c**) bar chart illustrating the distribution of the study sample by language skills profile; (**d**) histogram illustrating the distribution of the study sample by Words and Pseudowords; (**e**) histogram illustrating the distribution of the study sample by Expression.

The descriptive data for this study are presented in Table 1 and Figure 1. Of the 213 children enrolled, most were categorized as having typical language skills (61.7%) and approximately one in three as having atypical language skills (38.3%).

### 3.1. Main Outcome: Correlation of Different Language Skills Profiles with the COVID-19-Related Time Period

#### Differences in Language Skills Profile

**Figure 2:**
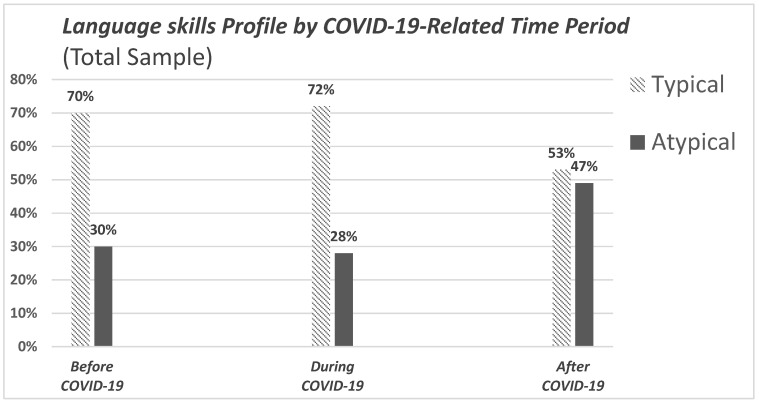
Bar chart illustrating the distribution of language skills profiles by COVID-19-related time period in the total study sample.

The distributions of children’s language skills profiles were tested among the three periods of time (pre, during and post COVID-19 pandemic) for the entire sample. The results showed a significant differentiation of the language skills profiles at these different periods [χ^2^ (2, N = 213) = 6.654, *p* = 0.036]. The proportion of children with an atypical profile is higher after the pandemic, when compared to the periods before and during the pandemic (Supplementary Table S1, Figure 2).

**Figure 3:**
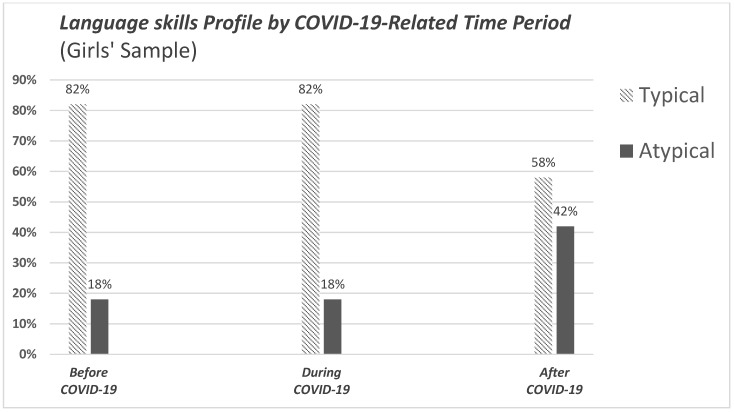
Bar chart illustrating the distribution of language skills profiles by COVID-19-related time period in the girls’ sample.

The above analysis was also performed separately between boys and girls as a sensitivity analysis. In the subgroup of boys, the chi-square test was not significant (*p* = 0.63). However, in the girls’ subgroup, the language skills profile was significantly associated with the period of measurement [χ^2^(2, N = 213) = 7.120, *p* = 0.028]. This analysis revealed that girls with an atypical language skills profile were found in a much higher proportion after the pandemic, compared to the proportions before and during the pandemic (Figure 3).

### 3.2. Secondary Outcome: Differences in Words/Pseudowords and Expression

**Figure 4:**
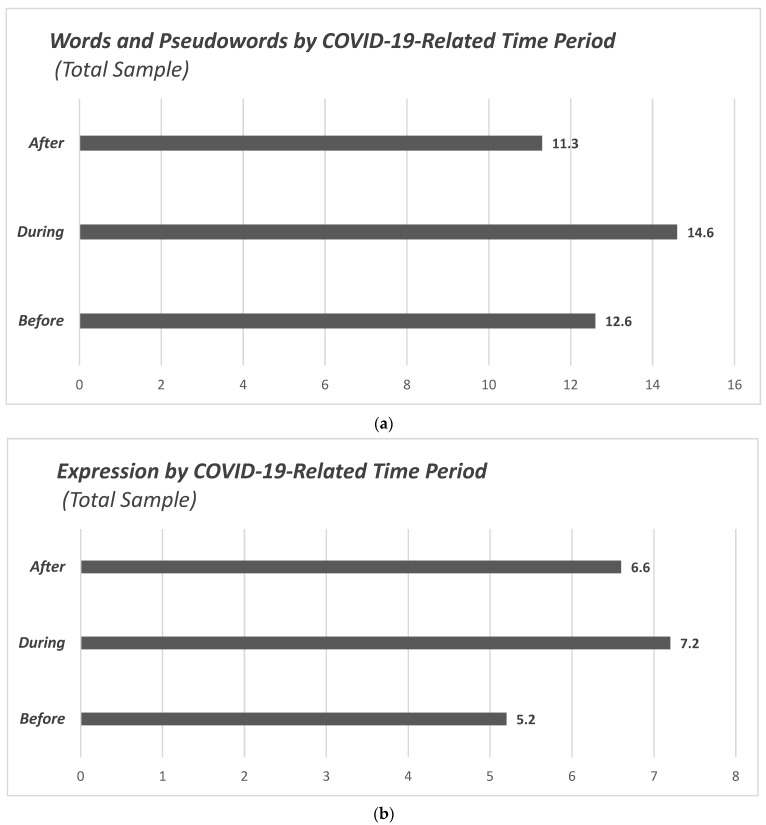
Bar charts illustrating the distribution of the sample across both Words/Pseudowords and Expression by COVID-19-related time period: (**a**) Words and Pseudowords by COVID-19-related time period (total sample); (**b**) Expression by COVID-19-related time period (total sample).

Additionally, within the same context of assessing language skills, it was examined whether the specific measurements in Words and Pseudowords, and in Expression had differences between the three periods (Figure 4). In the entire sample, the difference in Words and Pseudowords between the three periods was found to be significant (F(2.205) = 9.34, *p* < 0.001). The highest measurements were recorded during the pandemic (M = 14.6, SD = 2.9, Ν = 50), followed by before (M = 12.6, SD = 3.5, Ν = 62) and after (M = 11.3, SD = 5.3, Ν = 101) the pandemic. Post hoc analysis showed that the significant differences were in the pairs of periods: during/before (*p* = 0.044) and during/after (*p* < 0.001).

The difference in expression measurements between the periods was also found to be significant (F(2.205) = 14.197, *p* < 0.001). Children measured during the pandemic had the highest mean expression values (M = 7.2, SD = 1.9, N = 50), followed by post-pandemic (M = 6.6, SD = 2.3, N = 101) and pre-pandemic measurements (M = 5.2, SD = 2, N = 62). Post hoc testing yielded significant differences for both the during/before (*p* < 0.001) and during/after (*p* < 0.001) pairs.

**Figure 5:**
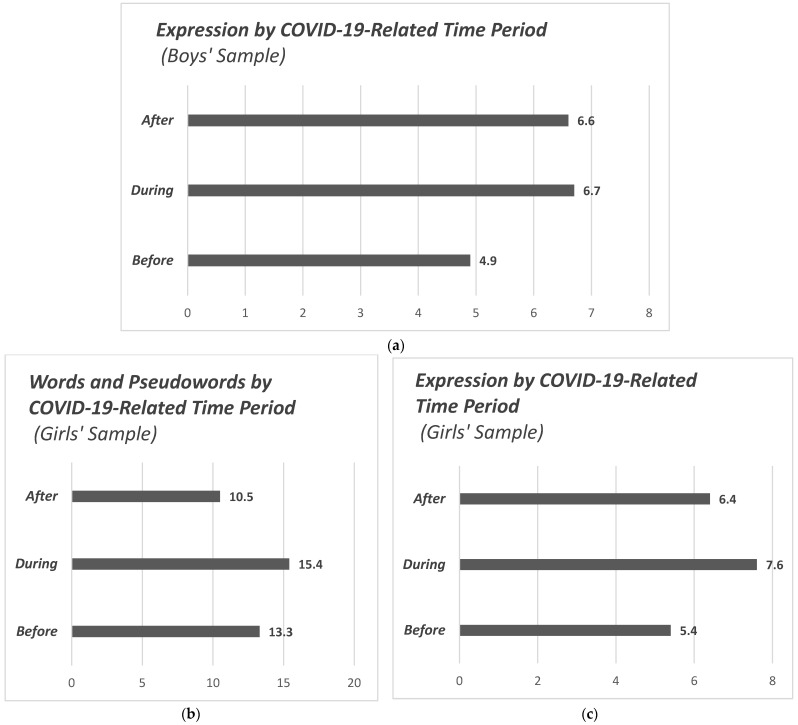
Bar charts illustrating the distribution of Words/Pseudowords and Expression in comparison of the two genders: (**a**) Expression by COVID-19-related time period (boys’ sample); (**b**) Words and Pseudowords by COVID-19-related time period (girls’ sample); (**c**) Expression by COVID-19-related time period (girls’ sample).

Additionally, the analysis above was performed separately between the two genders (Figure 5). In the sample of boys, no significant difference was found in Words and Pseudowords among the three periods (*p* = 0.70). In contrast, the differences in expression measurements were found to be significant (F(2.99) = 7.971, *p* = 0.001). Boys had higher expression scores mid pandemic (M = 6.7, SD = 2.2, Ν = 50), as well as post pandemic (M = 6.6, SD = 1.9, Ν = 101), and significantly lower scores pre pandemic (M = 4.9, SD = 2, Ν = 62). Post hoc testing identified significant differences between the before/during (*p* = 0.006) and before/after (*p* = 0.001) periods.

In the girls’ sample, Words and Pseudowords had significant differences in terms of period (F(2.103) = 10.958, *p* < 0.001). The highest values in girls were recorded during the COVID-19 pandemic (M = 15.4, SD = 1.2, Ν = 50), followed by values before (M = 13.3, SD = 3.5, Ν = 62) and after (M = 10.5, SD = 5.9, Ν = 101). Post hoc testing showed that significant differences are found between the before/after (*p* = 0.033) and during/after (*p* < 0.001) periods. Lastly, girls also recorded significant differences in Expression between the three periods (F(2.103) = 7.418, *p* = 0.001). The highest mean Expression scores were measured in girls during the COVID-19 pandemic (M = 7.6, SD = 1.4, Ν = 50), followed by measurements after (M = 6.4, SD = 2.6, Ν = 101) and before the pandemic (M = 5.4, SD = 2, Ν = 62). The post hoc test highlights as the only significant result the difference in the before/during periods (*p* = 0.001).

## 4. Discussion

The overall objective of this study was to evaluate the impact of the COVID-19 pandemic on the language skills of preschool children in Greece, taking into account their age and biological gender. This evaluation was conducted retrospectively through a physical assessment of their developmental language status, utilizing a screening tool and quantitative measurements of words, pseudowords and expressive language.

Of the total sample, one in three children appeared to have an atypical language skills profile (38.3%). In terms of the distribution of children’s profiles analyzed across three evaluation periods, our results indicated a significant association between language skills profile and measurement period. The atypical profile was significantly more frequent following the pandemic compared to the periods before and during the pandemic. The gender-related subgroup analysis revealed that boys exhibited no statistically significant differences in atypical language skills profiles across the three specified time periods. In contrast, girls demonstrated a marked increase in the prevalence of atypical language skills profiles in the post-pandemic period.

During the pandemic, a notable increase in the abundance of words and pseudowords, and modes of expression was observed in the total sample. In the boys’ subgroup, a statistically significant difference was identified solely in modes of expression during the pandemic. In contrast, girls demonstrated a significant increase not only in their expression but also in the usage of words and pseudowords during the same period.

Previous studies have also shed light on the impact of the pandemic on preschool children’s language development. Murillo et al. (2023) observed that pandemic-related factors negatively impacted language development in children during their first two years [16]. Erbay and Tarman (2022) concluded that reduced social interactions during the pandemic adversely affected children’s verbal language and social communication skills [17]. Additionally, Rodriguez-Rubio et al. (2023) conducted a study on Mexican toddlers, revealing that limited peer socialization during lockdowns led to delayed language development in post-pandemic children as compared to pre-pandemic cohorts [28]. Pejovic et al. (2024) and Feijoo et al. (2023) found that children born during the COVID-19 pandemic exhibited delays in early language (vocabulary and morphosyntactic) development compared to those born before it, with these effects persisting over time [18,19].

These studies collectively indicate that the COVID-19 pandemic has had a detrimental effect on early language development in preschool children, which aligns with our study outcomes and with the authors’ expectations. Preschool years are crucial for development across all domains, and recent research indicates that pandemic-related changes such as mask-wearing, peer isolation, and reduced social interactions and exposure to language-rich environments negatively impacted vocabulary acquisition and expressive language skills [9,12,20]. Infants represent the age group most vulnerable to the broader impacts of the pandemic. Social interaction, particularly with peers, and the ability to see facial expressions are vital for understanding language. This underscores the importance of participation in childcare settings; it was shown to enhance children’s language and executive function growth during the COVID-19 pandemic. This indirectly explains findings from various studies, including our own, revealing post-COVID-19 pandemic declines in language scores and impairments in motor and cognitive development of young students [9,12,14,15,23,24,25]. Another significant consequence of the pandemic measures is the increase in screen time, which has been significantly associated with poorer language development outcomes and higher risks of difficulties in both language comprehension and expressive skills [29]. Measures that hinder children’s ability to observe and imitate facial expressions, interpret emotional cues and regulate their emotions contribute to the emergence of developmental language disorders. These disorders adversely impact children’s communication skills, potentially hindering their social adaptation and overall life satisfaction [18,29,30,31].

Regarding children’s mental health, the WHO reports that the COVID-19 pandemic has markedly elevated global anxiety and depression, especially in children. The closure of childcare centers intensified stress for parents, particularly mothers, leading to increased maternal depression. This rise in maternal depression adversely affected preschoolers’ behavior. Research highlights the critical importance of positive mother/child interactions for children’s well-being, noting that interactions with depressed mothers can significantly raise the risk of early childhood depression [29,31,32,33,34]. Negative family environments, coupled with child temperament and stressful life events, appear to form a particularly detrimental combination that contributes to adverse outcomes in children’s mental health [31]. This may explain why children’s developmental profiles continued to show concerning trends even after pandemic-related restrictions were lifted, mirroring patterns seen in other crises (e.g., the war in Ukraine and rising inflation); adverse circumstances contribute to persistently high levels of parenting stress. The mental health of young children is closely tied to the psychosocial well-being of their caregivers [35,36,37,38].

In light of these considerations, a critical objective is to systematically evaluate the extent to which these changes affect children’s overall mental health and language development, as well as their quality of life. Additionally, it is essential to identify effective interventions to address these challenges [39]. Future research should investigate the underlying causes of all these observed trends and specifically the longitudinal relationship between parental well-being and child outcomes. Meanwhile, health experts must draw attention to the long-term effects of the pandemic on language trajectories and investigate whether children from different socioeconomic backgrounds were differentially affected. Thereby, guidance can be provided on minimizing potential impacts in similar emergency situations. Support interventions must prioritize fostering a positive family environment through conflict resolution strategies, reducing parenting stress and enhancing parental resources. This approach is critical for ensuring that caregivers can effectively address the needs of their young children, promoting not only typical language development—which is central to our study—but also supporting their overall physical and mental development, which remains the primary objective [4,18,30,31,34,40,41].

## 5. Limitations

We can categorize the limitations of this study into two main areas: those related to this study’s methodology and sample characteristics, and those pertaining to the process of assessing children’s language skills.

First, it is important to emphasize that this is a retrospective study with a relatively limited number of participants. Study participants were not the same at each time point, which may affect the consistency of our results. There was no follow-up on the progression of language development in the same sample over time. Although the families participating in the three COVID-19-related time periods exhibited similar sociodemographic characteristics, the results do not provide intra-individual observations from the same sample. Additionally, the sample was drawn from specific regions in Northern Greece, which may not be representative of the entire country. Adjustments for potential confounding factors that may adversely affect child development were also not conducted. These factors include, for example, any underlying neurodevelopmental disorders and disorder severity, COVID-19 infection or vaccination, as well as varying levels of preschool attendance, parental education and socioeconomic challenges that may exist independently of and simultaneously with the pandemic. Notably, both Central and Western Macedonia experience significant socioeconomic disparities, with rural areas facing fewer opportunities compared to urban centers. Hence, future studies could employ longitudinal or matched-sample designs to better isolate time period effects from sample composition differences.

Furthermore, the conditions under which the assessments were carried out raise questions about the accuracy of the language skills evaluations. The screenings took place in school settings, which are primarily designed for education and social interaction. This environment may have led to distractions for both the children and the evaluators, potentially compromising the integrity of the assessments. Additionally, while the “AνOμιΛο4” test has been specifically validated for children aged from 3.6 to 4.9 years old, it has been proven applicable up to 7 years old [42,43]. Given that our sample included children aged from 2.9 to 6.4 years, the test’s sensitivity and specificity may not be fully optimized for younger children (i.e., 2.9–3.5 years). However, the therapists at our center adapted the test questions to align with each child’s comprehension abilities based on their clinical experience. While this adaptation does not entirely eliminate the risk of misleading findings, the test was utilized to evaluate its practical applicability in task-based assessments of language production and processing in an age group that has not been previously studied.

## 6. Conclusions

Overall, our study uncovered a statistically significant difference in the language skills of preschool-aged children in Greece related to the COVID-19 pandemic, with one in three children of the total sample exhibiting an atypical language development profile. This trend was even more pronounced in the subsample collected after the pandemic, where 42% of children exhibited an atypical profile. Girls were mostly affected, and there was also an observed increase in the (re)production of words, pseudowords and modes of expression, particularly during the pandemic, a finding that warrants further investigation. The measures implemented during the pandemic seem to have had a substantial impact on the language skills and possibly on the language development of the children, both directly through restrictions imposed on children and indirectly by destabilizing adult caregivers, thereby affecting the overall well-being of their children.

## Figures and Tables

**Table 1:** Descriptive statistics (N = 213).

Questions	N (%) or M ± SD
Gender	
Boys	104 (48.8%)
Girls	109 (51.2%)
COVID-19-Related Time Period	
Before COVID-19 (% boys)	62 [52% boys]
During COVID-19 (% boys)	50 [44% boys]
After COVID-19 (% boys)	101 [49% boys]
Profile	
Typical	121 (61.7%)
Atypical	75 (38.3%)
Age (months)	
Total sample	53.9 ± 10.2
Before COVID-19	45.2 ± 6.5
During COVID-19	55.8 ± 9.6
After COVID-19	60.8 ± 7.6
Words and Pseudowords	12.5 ± 4.5
Expression	6.3 ± 2.2

M ± SD refers to mean ± standard deviation of the continuous variables of interest.

## Data Availability

The data that support the findings of this study are not publicly available due to privacy reasons but can be obtained from the corresponding author [A.K.] upon reasonable request.

## Supplementary Materials

The following supporting information can be downloaded at https://www.mdpi.com/article/10.3390/children12030376/s1: Table S1: Frequencies of the variables Language skills profile and COVID-19-related time periods in the total sample; Table S2: Frequencies of the variables Language skills profile and COVID-19-related time period in the girls’ sample; Table S3: Examples of questions asked during the test administration per task and area assessed.
