# Supplementary material for: The Impact of COVID-19 on the Language Skills of Preschool Children: Data from a School Screening Project for Language Disorders in Greece
Source: Children (Basel). 2025 Mar 18;12(3):376. doi: 10.3390/children12030376 (PMC11941272; doi:10.3390/children12030376)
Supplement: Supplementary file 1 [file children-12-00376-s001.zip › children-3497324-supplementary.pdf]

Supplementary Materials

Supplementary Table S1. Frequencies between the variables language skills profile and COVID-19 related time-period in the total sample.

	Before Covid	During Covid	After Covid
Typical	32	36	53
Atypical	14	14	47

Supplementary Table S2. Frequencies between the variables Language skills profile and COVID-19 related time-period in the girls' sample.

	Before Covid	During Covid	After Covid
Typical	18	23	30
Atypical	4	5	22

Supplementary Table S3. Examples of questions asked during the test administration per task and area assessed.

Area assessed	***Task***	***Response:***
Phonological processing (**Words**)	The examiner asks the child to name pictures or repeat words after them.<br>**Example**:<br>Do you see these pictures? What is this? A banana, a key, glasses, a vase, snow,closet, etc.?	The child names the pictures or repeats the words accurately.
Phonetic and articulatory skills of the child<br>Auditory perception<br>Short-term memory<br>(**Pseudowords**)	The examiner asks the children to repeat sequences of meaningless syllables (pseudowords)<br>**Example**:<br>Look at these Native Americans. They have strange names: Listen! This one is called "Hofa," "Vleggosa," "Vgoni," etc.	The child attempts to repeat the pseudowords accurately.

Encode an image.<br>Morphosyntax -Grammar and Sentence Structure<br>Lexical and Semantic Skills-Vocabulary and Meaning<br>Comprehension<br>Short-term memory<br>(**Expression**)	The examiner asks the child to respond to the questions being posed or to repeat a sentence<br>**Example**:<br>Look at this picture. This is John, and this is Mary. What do you see? How many children are in the picture? What is the boy doing, etc.?<br>I will tell you a sentence for you to say as well:<br>Tina ate the food.	The child attempts to produce or repeat age-appropriate sentences.
